# The Effect of Cooling Conditions on Martensite Transformation Temperature and Hardness of 15% Cr Chromium Cast Iron

**DOI:** 10.3390/ma13122760

**Published:** 2020-06-18

**Authors:** Mirosław Tupaj, Antoni Władysław Orłowicz, Andrzej Trytek, Marek Mróz, Grzegorz Wnuk, Anna Janina Dolata

**Affiliations:** 1 Faculty of Mechanics and Technology, Rzeszow University of Technology, Kwiatkowskiego 4, 37-450 Stalowa Wola, Poland; trytek@prz.edu.pl; 2 Department of Foundry and Welding, Rzeszow University of Technology, Al. Powstańców Warszawy 12, 35-959 Rzeszów, Poland; zois@prz.edu.pl (A.W.O.); mfmroz@prz.edu.pl (M.M.); gwnuk@prz.edu.pl (G.W.); 3 Department of Advanced Materials and Technologies, Faculty of Materials Engineering, Silesian University of Technology, Krasińskiego 8, 40-019 Katowice, Poland

**Keywords:** high-chromium cast iron, austenitizing conditions, cooling conditions, martensite transformation, hardness

## Abstract

The research reported in the paper concerned the conditions of cooling high-chromium cast iron with about 15% Cr content capable to ensure completeness of transformation of supercooled austenite into martensite in order to obtain high hardness value of the material and thus its high resistance to abrasive wear. For testing, castings were prepared with dimensions 120 mm × 100 mm × 15 mm cast in sand molds in which one of cavity surfaces was reproduced with chills. From the castings, specimens for dilatometric tests were taken with dimensions 4 mm × 4 mm × 16 mm and plates with dimensions 50 mm × 50 mm × 15 mm for heat treatment tests. The dilatometric specimens were cut out from areas subject to interaction with the chill. The austenitizing temperature and time were 1000 °C and 30 min, respectively. Dilatograms of specimens quenched in liquid nitrogen were used to determine martensite transformation start and finish temperatures T_Ms_ and T_Mf_, whereas from dilatograms of specimens quenched in air and in water, only T_Ms_ was red out. To secure completeness of the course of transformation of supercooled austenite into martensite and reveal the transformation finish temperature, it was necessary to continue cooling of specimens in liquid nitrogen. It has been found that T_Ms_ depended strongly on the quenching method whereas T_Mf_ values were similar for each of the adopted cooling conditions. The examined cooling variants were used to develop a heat treatment process allowing to obtain hardness of 68 HRC.

## 1. Introduction

Resistance to abrasive wear is one of the main criteria in the process of selection materials for construction and operation of machine components and parts [1]. Examples of such elements are replaceable inserts for dies used to form die stampings of refractory materials. The stampings are press molded of an aggregate of Al_2_O_3_-MgO type, containing also TiO_2_, SiO_2_, CaO, and Fe_2_O_3_. Particles of the aggregate, characterized by sharp corner edges and hardness values of about 1800 HV0.5, scratch surfaces of die inserts in the course of the press forming process. To date, the inserts are fabricated from tool steels thermally processed to hardness of up to 60–61 HRC. However, the service live of the inserts remains unsatisfactory to manufacturers of refractory materials.

One material used widely for components working in conditions of intensive abrasive wear specific for mining, ceramic, cement, and aggregate processing industries is the high-chromium cast iron [2,3,4,5]. Shaping high-chromium cast iron microstructure in order to improve resistance to abrasive wear is oriented at refinement of carbides by increasing the crystallization rate [6,7,8], improving hardness by introduction of alloying additions [9,10,11], and applying heat treatment [12,13] aimed at obtaining martensitic matrix [14,15,16]. However, too high martensite content may lead to fracturing of the matrix in the surface region. On the other hand, during impact loads, iron with a martensitic matrix is better in comparison to iron with the dominant austenitic matrix. The effect of microstructure in high stress abrasion of white cast irons (WCI) have been widely discussed by Heino et al. [17]. They concluded that the austenite-to-martensite ratio and also carbides structure strongly effect on the abrasion wear resistance of WCI specimens. 

Unpublished results of present author’s tests performed on plate castings of 15% Cr cast iron indicate that quenching them in air or in water from temperature of 1000 °C after austenitizing for 30 min is favorable from the point of view of high hardness values. 

Technical literature of the subject lacks results of research concerning the effect of quenching method on martensite transformation start and finish temperature, although such knowledge is necessary for the purpose of developing thermal treatment schedules securing completeness of transformation of supercooled austenite into martensite which would not produce excessive quenching stresses and the resulting hardening cracks. 

It is a well-known fact that the reduction of carbon content in austenite results in a decrease of the martensite transformation temperature. The effect of alloying elements on transformations of supercooled austenite in high-chromium cast iron results on their content in austenite. If complete dissolution of carbides was not achieved in the course of austenitizing, then content of carbon and alloying elements in austenite is lower than this revealed by chemical composition analysis of the alloy.

Value of the temperature T_Ms_ can be estimated based on chemical composition with the use of empirical formulas of the type proposed by author of the paper [18]. Relevant relationships are developed for conditions in which complete dissolution of carbides in austenite has occurred. The formulae indicate that the strongest effect on decrease of T_Ms_ value is produced by carbon, and further manganese, nickel, chromium, and molybdenum. Generality of these relationships is not sufficient and for that reason, dilatometric tests are carried out to determine T_Ms_ values more precisely. Dilatometry is a widely used experimental technique for measuring transformations and kinetics of solid-state phase transformations that involve dimensional changes. The course of martensite transformation is accompanied by changes in volume of specimens which are the base on which temperatures and kinetics of phase transformations can be determined with the use of the dilatometric method [19,20,21].

In view of the fact that carbides are characterized with the higher content of carbon and other alloying elements compared to austenite from which they precipitated, it can be claimed that in case of dissolution of carbides austenite is enriched in these elements. As a result, a decrease of T_Ms_ value is observed. On the other hand, presence of carbides on boundaries of austenite grains hampers their growth by hindering migration of grain boundaries. To break the grain boundary away from a carbide, an amount of energy must be supplied, for instance by heating the alloy up to a sufficiently high temperature. In the case of retention of fine austenite grains, conditions are created for rapid nucleation and development of such diffusional processes as eutectoid or bainitic transformation. It the applied cooling rate enables partial transformation of supercooled austenite into pearlite or bainite, that will be reflected in chemical composition of austenite and thus also in T_Ms_ value. As a result, T_Ms_ value decreases.

The issue of the effect of cooling rate on T_Ms_ in the process of quenching carbide-containing steels was discussed in a number of publications. Authors of [22] have found that in the case of low-alloy high-strength steels, lower values of T_Ms_ corresponded to higher specimen cooling rates. They assumed that at lower values of the cooling rate, precipitation of chromium and molybdenum carbides become possible. That resulted of impoverishment of austenite in content of carbon and alloying elements and ultimately in higher T_Ms_ values. In addition, authors of [23] have found that with increasing rate of cooling 9Cr-1.7W-0.4Mo-Co steel in the quenching treatment, a decrease of T_Ms_ value is observed. The authors suggest that the steel is susceptible to precipitation of carbides in austenite at low cooling rates which is reflected in impoverishment of its content of carbon and other alloying elements. At higher cooling rate values, the process of precipitation of carbides is blocked and austenite is no longer impoverished in carbon and other alloying elements. As a result, lower *T*_Ms_ values are observed. Authors of papers [24,25] suggest that by deformation of material in the course of austenitizing, it is possible to obtain increased density of lattice imperfections which supports nucleation of martensite and is favorable to increase of T_Ms_ value.

In paper [26] it was demonstrated that plastic deformation of steel at the austenitizing temperature resulted in an increase of T_Ms_ and broadening the T_Ms_–T_Mf_ temperature range. The authors of the study suggest that the effect is connected with an increase of the number of lattice defects that can act as nucleation sites for the start of the martensite transformation.

In the technical literature concerning the issue of high-chromium cast iron heat treatment, numerous research projects were devoted to the possibility to shape microstructure favorable for machinability or high resistance to abrasive wear of castings. Such studies are of great importance to the industrial practice.

Conventional quenching of high-chromium cast iron does not guarantee complete transformation of supercooled austenite into martensite which results in presence of retained austenite in the alloy matrix. It is known that at high chromium content values, retained austenite is stable up to the temperature range of 400–500 °C. Even longer soaking does not result in its transformation but is favorable to precipitation of carbides. That way an impoverishment in carbon and chromium occurs which, in the course of rapid cooling, facilitates transformation of austenite into martensite. In high-alloy steels rich in chromium, such heating and cooling cycle is repeated a number of times in order to secure complete transformation of retained austenite into martensite. Studies on thermal treatment of that type for high-chromium cast iron aimed at increase of its hardness were carried out by authors of papers [27,28,29,30,31]. A common feature of these reports consists of concluding that as a result of precipitation of secondary carbides, impoverishment of austenite in carbon and carbide forming alloying elements (Cr, Mn, Mo) occurs which leads to a rise in the martensite start T_Ms_ temperature. Therefore, the supercooled austenite in the course of quenching and the retained austenite in the course of tempering and rapid cooling can easily be transformed into martensite, resulting in bulk hardness and wear resistance increase.

Studies on development of new heat treatment schedules for high-chromium cast iron were conducted by authors of papers [32,33,34,35]. From this point of view, significant are studies on working out TTT (Time – Temperature – Transformation) plots serving as a base for selection of heat treatment parameter values [35].

Authors of [33] demonstrated the possibility to reduce hardness of high-chromium cast iron (14.55% Cr) castings with the use of soaking at subcritical temperatures or with the use of soaking at subcritical temperature preceded with quenching in oil. In the first case, hardness was reduced to the value 38.5 HRC and in the second instance, to 37 HRC whereas the hardness of the material in as-cast condition was 44 HRC. Such treatment was favorable to formation of the matrix microstructure containing “ferrite + grainy carbides”. Duration the first heat treatment variant was 31 h and was longer compared to the duration of the second variant which lasted for 17 h. Subcritical soaking temperatures were 650 °C and 750 °C in the first variant of the treatment and 725 °C in the second variant. For a high-chromium cast iron with the same chemical composition (14.55% Cr), the study [35] presented a heating dilatogram from which it followed that the eutectoid transformation start temperature was TAc_1_^p^ = 760 °C. Assuming that the rate of heating for which the dilatogram was taken corresponded to the heating rate applied in both of the softening treatment variants, an opportunity appeared for further decrease of hardness at shorter treatment cycle. That can be achieved by increasing the annealing temperature up to the range 725–760 °C.

Authors of [33] reported that in the case of high-chromium cast iron containing 2.7% C, 14.55% Cr, 2.20% Mn, and 0.93% Ni, austenitizing at temperature 950 °C for the period of 2 h secured precipitation of secondary carbides, mainly M_7_C_3_, and the following cooling in oil enabled partial transformation of supercooled austenite into martensite. A factor beneficial for the course of the transformation was a decrease of carbon and chromium content in austenite due to emergence of secondary carbides which led to an increase of T_Ms_ value. The volume fraction of non-transformed austenite was 64.9%. The cast iron with such structure was characterized by hardness of 62 HRC. So high hardness value could be made even higher by means of two methods promoting transformation of retained austenite into martensite. The first method would consist of the application of a one-time or multiple tempering in order to precipitate carbides from retained austenite and force the transformation of austenite into martensite with the use of rapid cooling. In the second method, cryogenic treatment could be used, aimed at transformation of retained austenite into martensite. Such approach to the issue is presented in this paper.

In view of the above, the objective of the study was to acquire a new knowledge in scope of the effect of cooling conditions from the adopted austenitizing temperature on martensite transformation limiting temperatures, and in particular the conditions securing completeness of transformation of supercooled austenite into martensite which is necessary to develop heat treatment processes securing low susceptibility to quenching cracks and high hardness of castings of chromium cast iron containing about 15% Cr.

## 2. Materials and Methods 

For the tests, chromium cast iron casting with about 15% content of Cr and dimensions 120 mm × 100 mm × 15 mm were made. Liquid metal was prepared in an induction furnace with about 20 kg charge capacity. The foundry mixture per 10 kg of alloy included: 4 kg of grade L210H21S cast steel; 1.5 kg of special pig iron LS; 0.2 kg of FeCr; 1.0 kg of steel; 0.08 kg of carburizer; 0.05 kg of FeMo; 0.03 kg of FeB; 0.05 kg of FeMn; 0.04 kg of FeSi; and 3 kg of scrap high-chromium cast iron. Analysis of chemical composition was carried out with the use of Q4 Tasman emission spectrometer (Bruker, Kalkar, Germany). Results of analysis are summarized in Table 1.

The liquid metal was poured at temperature 1450 °C into two twin-cavity sand molds with horizontally oriented cavities lower surfaces of which were reproduced by steel chills with thickness of 15 mm. Surfaces of chills were covered with black from acetylene-oxygen flame. The molds were provided with knife gates.

After shaking out from molds and cutting gate assemblies off, surfaces of the castings were cleaned in a jet of carborundum particles, smoothed by grinding, and cut into cubes with dimensions 60 mm × 50 mm × 15 mm using Struers Labotom-3 water-cooled metallographic cutter (Struers, Copenhagen, Denmark). Hardness of the cubes on the side of surfaces reproduced by chills was 58 HRC, and on the side of surfaces reproduced by sandmix was 48 HRC. From one side of the cubes, five slices of the material were cut off, each with thickness of 4 mm. Out of the slices, from the regions on the side of surface reproduced by the chill, specimens for dilatometric tests (Ø4 mm × 16 mm) were cut out with the use of BP95d wire erosion machine (ZAP B.P., Końskie, Poland )

Dilatometric tests were carried out on an upgraded dilatometer LS4 (Institute for Ferrous Metallurgy, Gliwice, Poland ) equipped with a computer program for furnace heating and cooling control as well as a program for recording elongation and temperature of specimens. Ni-CrNi thermocouples were used with wire diameter of 0.15 mm. The specimens were tested in protective atmosphere of argon. The specimens were heated at rate V_heat_ = 400 °C/h up to and maintained at the austenitizing temperature of 1000 °C for 30 min and then cooled in compressed air, water, or liquid nitrogen.

The analysis of microstructures was performed using a VEGA 3 scanning electron microscope (SEM, TESCAN, Brno, Czech Republic). All prepared samples were etched in Kalling’s reagent for matrix/carbide contrast. The volume fraction of carbide was evaluated with the use of Neophot 2 optical microscope equipped with Videotronic CC20P camera (Videotronic International, Rastatt, Germany) and Multiscan v.08 advanced image analysis system (University of Warsaw, Warsaw, Poland). The analysis was carried out in 10 areas under 500× magnification. 

## 3. Results and Discussion 

**Figure 1:**
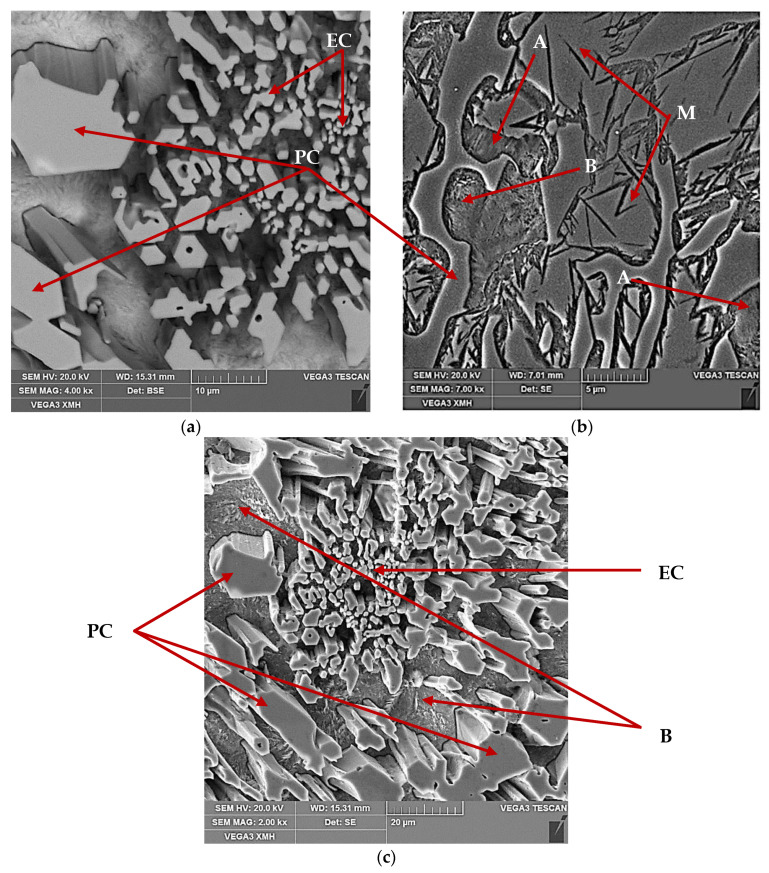
SEM microstructure of 15% Cr cast iron specimen as cast condition. Different phases, primary chromium carbide precipitates (PC), eutectic carbide precipitates (EC), martensite (M), bainite (B) and austenite (A) are indicated by arrows: (**a**) after deep etching, (**b**) after light etching, (**c**) after deep etching.

The characteristic microstructure of 15% Cr cast iron in its original state is shown in Figure 1. Microstructure of the cast iron in the as-cast condition is characterized by primary carbides and the matrix containing bainite, the austenite-eutectic carbides eutectic, and martensite. The volume fraction of carbides fell into the range 29.2–30.7%.

**Figure 2:**
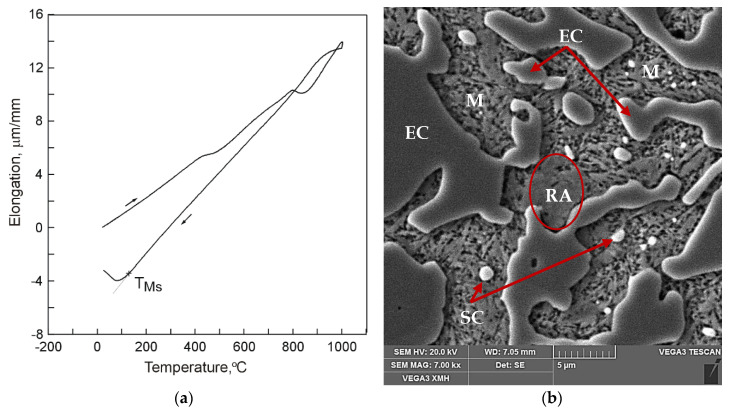
The 15% Cr cast iron specimen (V_heat_ = 400 °C/h, T_A_ = 1000 °C, τ_A_ = 30 min) cooled in air (V_cool 800–400_ = 6.9 °C/s, V_cool 400–TMs_ = 1.9 °C/s): (**a**) a dilatogram; (**b**) SEM microstructure, etched section; retained austenite (RA), martensite (M), eutectic carbides (EC), and secondary carbides (SC) have been marked.

**Figure 3:**
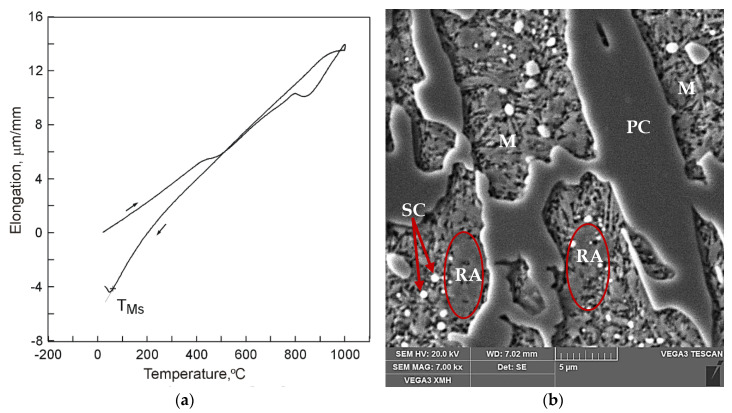
A dilatogram for 15%Cr cast iron (V_heat_ = 400 °C/h, T_A_ = 1000 °C, τ_A_ = 30 min) cooled in water (V_cool 800–400_ = 20.9 °C/s, V_cool 400–TMs_ = 3.8 °C/s): (**a**) a dilatogram; (**b**) SEM microstructure, etched section; retained austenite (RA), martensite (M), primary (PC), and secondary carbides (SC) have been marked.

**Figure 4:**
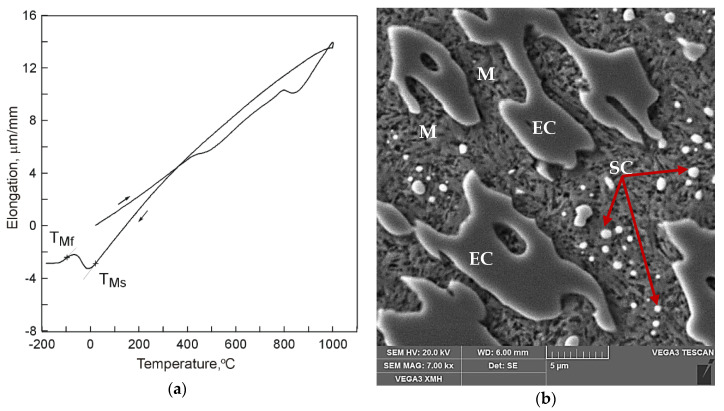
A dilatogram for 15% Cr cast iron (V_heat_ = 400 °C/h, T_A_ = 1000 °C, τ_A_ = 30 min) cooled in liquid nitrogen (V_cool 800–400_ = 14.8 °C/s, V_cool 400–TMs_ = 6.0 °C/s): (**a**) a dilatogram; (**b**) SEM microstructure, etched section; martensite matrix (M) as well as eutecitc (EC), and secondary carbides (SC) have been marked.

Example dilatograms and selected SEM microstructure images for the different states of the treated samples are presented in Figure 2, Figure 3 and Figure 4. Martensite transformation start and finish temperatures were determined with the use of the tangent method. The dilatograms contain also average values of the cooling rate in temperature ranges 800–400 °C and 400 °C–TMs.

It has been found that the course of martensite transformation was accompanied by an increase of cast iron volume which can be noted on dilatograms in the form of inflection in the direction of increasing specimen elongation values with decreasing temperature (Figure 2, Figure 3 and Figure 4). Once its driving force in the form of decreasing temperature was used up, the transformation of supercooled austenite into martensite was halted which was observed in case of cooling in compressed air (Figure 2a) and in water (Figure 3a). As a result, residual austenite was still visible in the cast iron microstructure. Representative microstructure images are shown in Figure 2b and Figure 3b. 

The use of cooling medium with much lower temperature, which was liquid nitrogen (−195.8 °C), resulted in occurrence of a full course of transformation of supercooled austenite into martensite (Figure 4). In the dilatogram of Figure 4a, that is evidenced by occurrence of an inflection in the direction of decreasing specimen elongation values. As can be seen in Figure 4b, the microstructure of the analyzed samples consisted of eutectic carbides (EC) and secondary carbides (SC) surrounded by a martensite matrix (M).

It is worth noting that in the first temperature range 800–400 °C, the cooling rate in liquid nitrogen is lower than the cooling rate in water. That is a result of turbulent evaporation of the medium around the specimen mounted in the dilatometer tube. In the second temperature range 400 °C–T_Ms_, intensity of evaporation of liquid nitrogen decreased significantly which improved effectiveness of heat sinking from specimens. Within that temperature range, the highest cooling rate was observed in the specimen quenched in liquid nitrogen, somewhat lower in the specimen hardened in water, and lowest in the specimen quenched in air. 

Results of examination of the effect of medium used to quench 15% Cr cast iron on cooling rate value in temperature ranges 800–400 °C and 400 °C–T_Ms_ and martensite transformation limiting temperatures are given in Table 2.

The obtained results indicate that cooling conditions have an effect on the martensite transformation start temperature. Hysteresis of the martensite transformation start temperature is characterized by decreasing together with increasing cooling rate in the second temperature range 400 °C–T_Ms_. For cooling conditions either in air, water, or liquid nitrogen, the martensite transformation start temperature assumes positive values, from 128 °C for air, through 62 °C for water, to 20 °C for liquid nitrogen. Only the use of liquid nitrogen secures completeness of transformation of supercooled austenite into martensite.

Examination of the effect of the cooling medium type (compressed air, water, liquid nitrogen) used in the process of quenching plate specimens on their hardness values were carried out for the same cooling rate values and conditions of austenitizing as those adopted in dilatometric tests. In the course of heating and austenitizing in Nabertherm N/61H furnace, specimens were dusted with dry quartz sand. Hardness was evaluated with the use of Rockwell hardness testing machine in scale C. Results of testing hardness of the specimens on the side reproduced by chill are summarized in Table 3.

The obtained results indicate that the use of liquid nitrogen as the cooling medium resulted in the temperature decrease deep enough to secure completeness of martensite transformation in the group of tested specimens. It turned out that cooling in water allowed to achieve higher specimen hardness values compared to those observed in specimens cooled in compressed air. The results suggest that the degree of transformation of supercooled austenite into martensite obtained in the course of cooling the specimens in water is higher compared to the degree of transformation of supercooled austenite into martensite in specimens cooled in compressed air.

**Figure 5:**
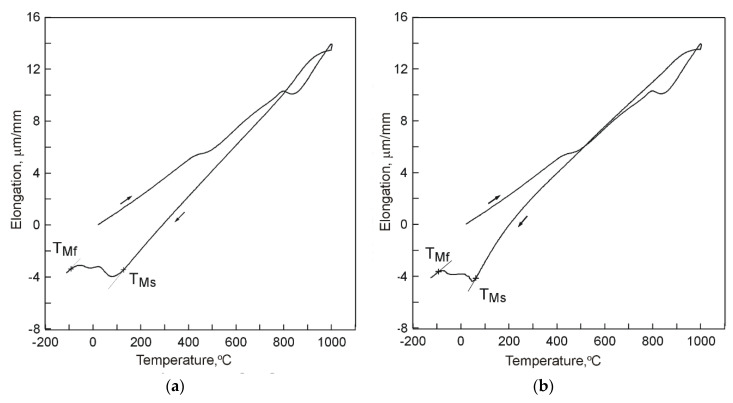
A dilatograms of 15% Cr cast iron (V_heat_ = 400 °C/h, T_A_ = 1000 °C, τ_A_ = 30 min) cooled: (**a**) in air and after 1 h, in liquid nitrogen; (**b**) in water and after 1 h, in liquid nitrogen.

Further dilatometric tests included two stages of cooling. After 1 h, the specimens cooled in compressed air were, without dismounting from dilatometer, subject to further cooling in liquid nitrogen (deep cryogenic treatment). The same dilatometric testing procedure was applied to specimens cooled in water. Example dilatograms taken in the course of the tests are presented in Figure 5.

**Figure 6:**
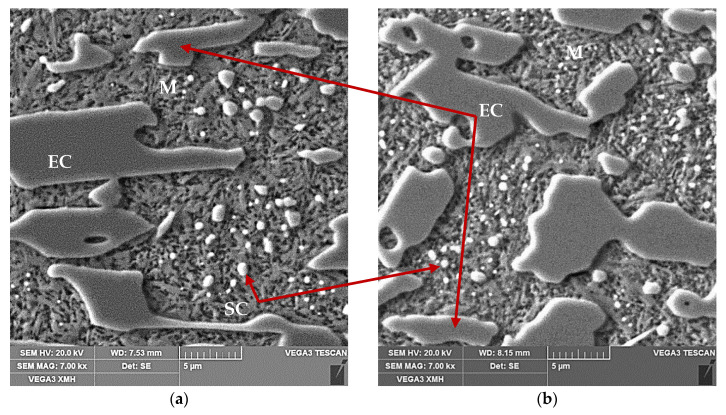
The SEM microstructure images of 15% Cr cast iron (V_heat_ = 400 °C/h, T_A_ = 1000 °C, τ_A_ = 30 min) cooled: (**a**) in air and after 1 h, in liquid nitrogen; (**b**) in water and after 1 h, in liquid nitrogen.

Analysis of the obtained dilatograms indicates that application of the two-stage procedure of cooling to dilatometric specimens triggered resumption of transformation of supercooled austenite into martensite (Figure 6).

The obtained dilatograms illustrate changes in specimen dimensions in the course of temperature decreasing. The nature of the changes is however different than in the case of direct cooling the specimens in liquid nitrogen (Figure 4). The last resolute inflection of the plot line in direction corresponding to decrease of specimen dimensions evidences occurrence of a complete course of supercooled austenite transformation into martensite and allows to determine the martensite transformation finish temperature T_Mf_. The obtained dilatograms evidence the possibility to trigger transformation of supercooled austenite into martensite as a result of immersing specimens in a cooling medium with sufficiently low temperature. The microstructure of the tested samples consisted of martensite (M), eutectic carbides (EC), and secondary carbides (SC), whereas no residual austenite (RA) was observed (Figure 6). On the other hand, it can be assumed that the course of transformation of supercooled austenite into martensite is less violent compared to the variant with direct cooling in liquid nitrogen. This should result in lower level of quenching stresses and thus less susceptibility of castings to quenching cracks.

Results of the research on the effect of two-stage quenching on martensite transformation temperatures in 15% Cr cast iron are given in Table 4.

The obtained results indicate that the use of a medium with significantly lower temperature such as liquid nitrogen (–195.8 °C) in the second stage of cooling allowed to finish off the process of transformation of supercooled austenite still remaining in specimens cooled in air or in water into martensite.

When two-stage cooling was applied in order to ensure completeness of martensite transformation, T_Mf_ values in the variant with compressed air and in the variant with water (−93 °C and −95 °C, respectively) may be considered comparable.

Results of examination of the effect of two-stage cooling first in compressed air and then in liquid nitrogen or first in water and then in liquid nitrogen on hardness values of plate specimens on their sides reproduced with chills are given in Table 5. The plate specimens, protected from oxidation by dusting with a dry quartz sand sprinkle, were austenitized at temperature 1000 °C for 30 min in Nabertherm N/61H furnace.

The obtained results indicate that under the same conditions of austenitizing, application of diversified processes of cooling securing complete transformation of supercooled austenite into martensite allowed to obtain the same high hardness values of 15% Cr cast iron.

The austenite, in view of its supersaturation with carbon and alloying elements, is thermodynamically unstable. For that reason, it is susceptible to precipitation of carbides out of it. Elevation of alloy temperature favors the process energetically. Precipitation of carbides in high-chromium cast iron leads to impoverishment of austenite in carbon, chromium, and other elements included in composition of chromium carbides. Authors of the paper [35] demonstrated that precipitations of chromium carbides from austenite in a cast iron containing 14.55% Cr proceeded fastest at the temperature of 950 °C. The process started as early as after 10 s and ended within the period of 160 min. The austenite impoverished in carbon and other alloying elements, transforms into martensite in the course of rapid cooling. The austenite-martensite transformation temperature is the higher, the less carbon and carbide formers are contained in austenite. The process of precipitation of chromium carbides requires diffusive migration of atoms making up the carbides to thermodynamically favorable locations such as grain boundary defects, crystalline lattice defects, glide bands, or austenite regions adjacent to carbides in which plastic deformations could occur as a result of different linear expansion coefficient values. The observed higher values of T_Ms_ for conditions of quenching in air can be explained so that the period of keeping specimens in the range of high temperatures turned out to be sufficiently long for the process of precipitation of carbides and change in chemical composition of austenite. In the conditions of cooling in liquid nitrogen, the period of maintaining specimens in the range of high temperatures was too short for the process of precipitation of carbides in the course of cooling which resulted in lower T_Ms_ values.

## 4. Conclusions

The obtained results of the research on the effect of variant cooling processes applied to 15% Cr cast iron specimens from the austenitizing temperature of 1000 °C after the austenitizing time of 30 min on martensite transformation temperatures and hardness values indicate that:

- the cooling rate, especially in the temperature range 400 °C–T_Ms_, is decisive for martensite transformation start temperatures;
- cooling in air or in water does not secure completeness of transformation of supercooled austenite into martensite;
- the martensite transformation start temperature T_Ms_ is sensitive to the cooling rate and amounts to 128 °C for cooling in air, 62 °C for cooling in water, and 20 °C for cooling in liquid nitrogen;
- in case of cooling in air or in water, it is possible to force completeness of the course of transformation of supercooled austenite into martensite, however this requires application of an additional operation of cooling in a medium with temperature lower than the martensite transformation finish temperatures (−93 °C and −95 °C, respectively);
- application of diversified variant cooling processes guaranteeing completeness of transformation of supercooled austenite into martensite allowed to obtain the hardness value of 68 HRC in plate specimens on their side reproduced by chills.

## Figures and Tables

**Table 1:** Chemical composition of 15% Cr cast iron.

Element Content (wt.%)										
C	Si	Mn	P	S	Cr	Mo	Ni	Cu	B	Fe
3.50	1.10	0.65	0.03	0.030	15.30	0.50	0.30	0.04	0.02	Bal

**Table 2:** The effect of type of medium used to quench 15% Cr cast iron on value of the cooling rate V_cool_ in temperature ranges 800–400 °C and 400 °C–T_Ms_ and on martensite transformation limiting temperature values.

Cooling Medium	Air	Water	Liquid Nitrogen
Cooling rate ( °C/s)			
in the range 800–400 °C	6.9	20.9	14.8
in the range 400 °C–T_Ms_	1.9	3.8	6.0
T_Ms_ ( °C)	128	62	20
T_Mf_ ( °C)	n.a.	n.a.	−97

* Heating rate V_heat_ = 400 °C/h; Austenitizing temperature T_A_ = 1000 °C; Austenitizing time τ_A_ = 30 min

**Table 3:** Hardness values for 15% Cr cast iron plate specimens on the side reproduced by chill, cooled in compressed air, water, or liquid nitrogen from temperature 1000 °C after austenitizing for 30 min.

Cooling Medium	Air	Water	Liquid Nitrogen
HRC hardness	63	64	68

* Heating rate V_heat_ = 400 °C/h; Austenitizing temperature T_A_ = 1000 °C; Austenitizing time τ_A_ = 30 min

**Table 4:** Results of the research on determination of martensite transformation limiting temperatures in 15% Cr cast iron after two-stage cooling, first in compressed air and then in liquid nitrogen and first in water and then in liquid nitrogen.

Cooling Medium	First Compressed Air, then Liquid Nitrogen	First Water, then Liquid Nitrogen
T_Ms_ ( °C)	128	62
T_Mf_ ( °C)	−93	−95

* Heating rate V_heat_ = 400 °C/h; Austenitizing temperature T_A_ = 1000 °C; Austenitizing time τ_A_ = 30 min

**Table 5:** Results of hardness measurements for plate specimens of 15% Cr cast iron austenitized at 1000 °C for 30 min, then cooled in compressed air and further in liquid nitrogen or in compressed air and further in liquid nitrogen. Measurements taken on surface reproduced by chill.

Cooling Medium	First Compressed Air, then Liquid Nitrogen	First Water, then Liquid Nitrogen
Plate hardness (HRC)	68	68

* Heating rate V_heat_ = 400 °C/h; Austenitizing temperature T_A_ = 1000 °C; Austenitizing time τ_A_ = 30 min

## References

[1] Hutchings I.M., Shipway P. (2017). Design and selection of materials for tribological applications. Tribology—Friction and Wear of Engineering Materials.

[2] Atapek S.H., Polat S.A. (2013). Study of wear of high-chromium cast iron under dry friction. Metal Sci. Heat Treat..

[3] Gasan H., Erturk F. (2013). Effects of a destabilization heat treatment on the microstructure and abrasive wear behavior of high-chromium white cast iron investigated using different characterization techniques. Metall. Mater. Trans..

[4] Gu J., Xiao P., Song J., Li Z., Lu R. (2018). Sintering of a hypoeutectic high chromium cast iron as well as its microstructure and properties. J. Alloys Compd..

[5] Pokusová M., Brúsilová A., Šooš L., Berta I. (2015). Abrasion Wear Behavior of High-chromium Cast Iron. Arch. Foundry Eng..

[6] Coronado J.J., Sinatora A. (2009). Abrasive wear study of white cast iron with different solidification rates. Wear.

[7] Gromczyk M., Kondracki M., Studnicki A., Szajnar J. (2015). Stereological Analysis of Carbides in Hypoeutectic Chromium Cast Iron. Arch. Foundry Eng..

[8] Orłowicz W., Trytek A. (2003). Effect of rapid solidification on sliding wear of iron castings. Wear.

[9] Scandian C., Boher C., Mello J.D.B., Rezai-Aria F. (2009). Effect of molybdenum and chromium contents in sliding wear of high-chromium white cast iron: The relationship between microstructure and wear. Wear.

[10] Studnicki A., Dojka R., Gromczyk M., Kondracki M. (2016). Influence of Titanium on Crystallization and Wear Resistance of High Chromium Cast Iron. Arch. Foundry Eng..

[11] Kopyciński D., Piasny S., Kawalec M., Madizhanova A. (2014). The Abrasive Wear Resistance of Chromium Cast Iron. Arch. Foundry Eng..

[12] Kopyciński D., Guzik E., Siekaniec D., Szczęsny A. (2014). Analysis of the High Chromium Cast Iron Microstructure after the Heat Treatment. Arch. Foundry Eng..

[13] Gonzalez-Pociño A., Alvarez-Antolin F., Asensio-Lozano J. (2019). Influence of Thermal Parameters Related to Destabilization Treatments on Erosive Wear Resistance and Microstructural Variation of White Cast Iron Containing 18% Cr. Application of Design of Experiments and Rietveld Structural Analysis. Materials.

[14] Coronado J.J., Gómez A., Sinatora A. (2009). Tempering temperature effects on abrasive wear of mottled cast iron. Wear.

[15] Hadji A., Bouhamla K., Maouche H. (2016). Improving Wear Properties of High-Chromium Cast Iron by Manganese Alloying. Int. J. Metalcast..

[16] Bedolla-Jacuinde A., Guerra F., Mejia I., Vera U. (2019). Niobium Additions to a 15%Cr–3%C White Iron and its Efects on the Microstructure and on Abrasive Wear Behavior. Metals.

[17] Heino V., Kallio M., Valtonen K., Kuokkala V.T. (2017). The role of microstructure in high stress abrasion of white cast irons. Wear.

[18] Andrews K.W. (1965). Empirical Formulae for the Calculation of Some Transformation Temperatures. J. Iron Steel Inst..

[19] Yang H.S., Bhadeshia H.K.D.H. (2007). Uncertainties in Dilatometric Determination of Martensite Start Temperature. Mater. Sci. Technol..

[20] Warke V.S., Sisson R.D., Makhlouf M.M. (2009). A Model for Converting Dilatometric Strain Measurements to the Fraction of Phase Formed During the Transformation of Austenite to Martensite in Powder Metallurgy Steels. Metall. Mater. Trans. A.

[21] Gomez M., Medina S.F., Caruana G. (2003). Modelling of Phase Transformation Kinetics by Correction of Dilatometry Results for a Ferritic Nb-microalloyed Steel. ISIJ Int..

[22] De Souzaa S.d., Moreiraa P.S., de Fariaa G.L. (2020). Austenitizing Temperature and Cooling Rate Effects on the Martensitic Transformation in a Microalloyed-Steel. Mater. Res..

[23] Gao Q., Wang C., Qu F., Wang Y., Qiao Z. (2014). Martensite transformation kinetics in 9Cr-1.7W-0.4Mo-Co ferrite steel. J. Alloys Compd..

[24] Durlu T.N. (2001). Effects of High Austenitizing Temperature and Austenite Deformation on Formation of Martensite in Fe-Ni-C Alloys. J. Mater. Sci..

[25] Sastri A.S., West D.R.F. (1965). Effect of Austenitizing Conditions on the Kinetics of Martensite Formation in Certain Medium-Alloy Steels. J. Iron Steel Inst..

[26] Alvarado-Meza M.A., García-Sanchez E., Covarrubias-Alvarado O., Salinas-Rodriguez A., Guerrero-Mata M.P., Colás R. (2013). Effect of the High-Temperature Deformation on the Ms Temperature in a Low C Martensitic Stainless Steel. J. Mater. Eng. Perform..

[27] Efremenko V., Shimizu K., Chabak Y. (2013). Effect of Destabilizing Heat Treatment on Solid-State Phase Transformation in High-Chromium Cast Irons. Metall. Mater. Trans. A.

[28] Powell G.L.F., Laird G.J. (1992). Structure, nucleation, growth and morphology of secondary carbides in high chromium and Cr-Ni white cast irons. Mater. Sci..

[29] Maratray F. (1971). Choice of appropriate compositions for chromium-molybdenum white irons. Trans. AFS.

[30] Wiengmoon A., Chairuangsri T., Pearce J.T.H. (2004). A Microstructural Study of Destabilised 30wt%Cr-2.3wt%C High Chromium Cast Iron. Iron Steel Inst. Jpn. Int..

[31] Laird G., Powell G.L.F. (1993). Solidification and Solid-State Transformation Mechanisms in Si Alloyed High-Chromium White Cast Irons. Metall. Trans. A.

[32] Guitar M.A., Suárez S., Prat O., Guigou M.D., Gari V., Pereira G., Mücklich F. (2018). High Chromium Cast Irons: Destabilized-Subcritical Secondary Carbide Precipitation and Its Effect on Hardness and Wear Properties. J. Mater. Eng. Perform..

[33] Efremenko V.G., Wu K.M., Chabak Y.U.G., Shimizu K., Isayev O.B., Kudin V.V. (2018). Alternative Heat Treatments for Complex-Alloyed High-Cr Cast Iron Before Machining. Metall. Mater. Trans. A.

[34] Karantzalis A.E., Lekatou A., Mavros H. (2009). Microstructural Modifications of As-Cast High-Chromium White Iron by Heat Treatment. J. Mater. Eng. Perform..

[35] Efremenko V.G., Chabak Y.U.G., Brykov M.N. (2013). Kinetic Parameters of Secondary Carbide Precipitation in High-Cr White Iron Alloyed by Mn-Ni-Mo-V Complex. J. Mater. Eng. Perform..

